# Multifeature analysis of an ultrasound quantitative diagnostic index for classifying nonalcoholic fatty liver disease

**DOI:** 10.1038/srep35083

**Published:** 2016-10-13

**Authors:** Yin-Yin Liao, Kuen-Cheh Yang, Ming-Ju Lee, Kuo-Chin Huang, Jin-De Chen, Chih-Kuang Yeh

**Affiliations:** 1 Department of Biomedical Engineering, Hungkuang University, Taichung, 43302 Taiwan; 2 Department of Community and Family Medicine, National Taiwan University Hospital Hsinchu Branch, Hsinchu, 30059 Taiwan; 3 Department of Family Medicine, National Taiwan University Hospital Bei-Hu branch, Taipei, 10845 Taiwan; 4 Department of Biomedical Engineering and Environmental Sciences, National Tsing Hua University, Hsinchu, 30013 Taiwan; 5 Department of Family Medicine, National Taiwan University Hospital, Taipei, 10048 Taiwan; 6 Department of Family Medicine, College of Medicine, National Taiwan University, Taipei, 10671 Taiwan; 7 Department of Internal Medicine, National Taiwan University Hospital, Bei-Hu branch, Taipei, 10845 Taiwan

## Abstract

Nonalcoholic fatty liver disease (NAFLD) is a chronic liver disease related to metabolic syndrome. This study applied an integrated analysis based on texture, backscattering, and attenuation features in ultrasound imaging with the aim of assessing the severity of NAFLD. Ultrasound radiofrequency data obtained from 394 clinical cases were analyzed to extract three texture features (autocorrelation, sum average, and sum variance), the signal-to-noise ratio (SNR), and the slope of the center-frequency downshift (CFDS slope). The texture, SNR, and CFDS slope were combined to produce a quantitative diagnostic index (QDI) that ranged from 0 to 6. We trained the QDI using training data and then applied it to test data to assess its utility. In training data, the areas (AUCs) under the receiver operating characteristic curves for NAFLD and severe NAFLD were 0.81 and 0.84, respectively. In test data, the AUCs were 0.73 and 0.81 for NAFLD and severe NAFLD, respectively. The QDI was able to distinguish severe NAFLD and a normal liver from mild NAFLD, and it was significantly correlated with metabolic factors. This study explored the potential of using the QDI to supply information on different physical characteristics of liver tissues for advancing the ability to grade NAFLD.

Nonalcoholic fatty liver disease (NAFLD) refers to a spectrum of disorders, ranging in severity from excess triglycerides as lipid-droplets accumulation in hepatocytes to steatohepatitis and eventually fibrosis, cirrhosis, and hepatocellular carcinoma^1^,^2^. Risk factors for NAFLD include obesity, hyperlipidemia, and insulin-resistance diabetes mellitus, and it may also represent another complication of metabolic syndrome^3^,^4^. NAFLD has become an important health issue because of its high prevalence, occurring in one in three persons in the developed world^2^,^5^. Nevertheless, NAFLD is a reversible condition, particularly during the early stages of the disease^6^. Diagnosing and staging patients with NAFLD are critical to preventing the development of an irreversible advanced liver disease.

Liver biopsy is the gold standard for NAFLD diagnosis and evaluation. Histological grading and staging are widely used in the assessment of liver biopsy samples obtained from patients with NAFLD^7^. The histological findings are graded as fatty change, ballooning, and inflammation, and the staging includes an assessment of the severity of fibrosis. However, performing a liver biopsy is invasive and can cause severe complications (e.g., infection, bleeding, and bile leakage)^8^. Serum biochemical tests provide information about the liver function and so are used as indicators of the metabolic status^9^,^10^. However, more than two-thirds of patients have normal liver function at any given time, and the liver function parameters are not correlated with histology findings and so are not helpful for diagnosing NAFLD^11^.

Several imaging techniques have also been used in the diagnosis of NAFLD. Computed tomography (CT) imaging of the liver is useful since NAFLD decreases the CT attenuation of the liver^12^,^13^. Three measures have been investigated: absolute measurements of attenuation in Hounsfield units, differences in attenuation between the liver and spleen, and the ratio of these values^14^. Magnetic resonance (MR) imaging and spectroscopy are considered reliable imaging methods for quantifying liver fat in the detection of NAFLD^15^,^16^. Fatty changes are assessed by differences in chemical shifts between fat and water; that is, the difference in MR frequency between the protons in fat and water^16^. However, how steatohepatitis and fibrosis influence the liver fat fraction is still unknown^17^. Even though MR and CT techniques have the ability to detect NAFLD with a high accuracy, MR techniques are relatively costly and time-consuming, and CT is associated with radiation exposure^2^. Therefore, these techniques are not extensively employed in the clinical setting and community-based epidemiology studies.

Ultrasound has been suggested to be an acceptable first-line screening tool for NAFLD due to its widespread availability and safety^18^,^19^. The different gray levels in an ultrasound image reflect acoustic properties of different tissue structures, such as the attenuation of acoustic waves, speed of sound, and acoustic impedance. NAFLD appears brighter in an ultrasound image relative to the adjacent kidney or spleen, and the attenuation is larger in severe cases, often obscuring the hepatic and portal vein walls^20^,^21^. The sensitivity and specificity of ultrasound for detecting NAFLD have ranged from 60–94% and 84–95%, respectively, in various studies, with the sensitivity being lower when the degree of NAFLD is mild^20^,^22^,^23^. Previous studies proposed different scoring systems for diagnosing NAFLD^24^,^25^,^26^. The system of Hamaguchi *et al*. is based on the histological Matteoni’s criteria, which were not originally used for an NAFLD diagnosis^25^. Furthermore, the system of Saadeh *et al*. is only useful when the fatty infiltration is >33%, while that of Liang *et al*. was developed based on morbidly obese patients^24^,^26^. Therefore, these scoring systems might not be appropriate for the general population due to either their original designs or poor performance. Ballestri *et al*. recently developed the ultrasound fatty liver index (US-FLI), which is a semiquantitative scoring system based on ultrasound findings^27^,^28^. This index is significantly positively correlated with the percentage of steatosis in histology (Pearson correlation coefficient = 0.745) and was developed based on histopathological Brunt’s criteria that have been suggested to be useful for clinical NAFLD diagnoses^28^.

Quantitative assessments of echo intensities have also been investigated with the aim of producing an objective method for the diagnosis of NAFLD^29^,^30^,^31^. Quantitative tissue characterization techniques can be divided into two main categories based on extracting parameters from either (1) B-mode images after echo processing or (2) backscattered ultrasound radiofrequency (RF) signals^29^. Because histograms display the occurrence frequency of gray levels, first, second, and run-length statistics—which give characteristic features called texture parameters of the B-mode image structure—have been used to classify liver abnormalities^32^,^33^,^34^. Backscattered ultrasound RF signals are dependent on the size, density, arrangement, and other properties of scatterers in the tissue^35^,^36^,^37^. Several studies have found that the increases in attenuation and backscatter coefficients as computed from RF echo data are larger in livers with NAFLD than in healthy livers^30^,^33^,^38^. However, most of these studies only distinguished between normal and pathological livers, and did not grade the severity of NAFLD. In addition, Acharya *et al*. considered that the diagnosis performance of ultrasound could be markedly improved by adding features extracted from the backscattered signals to those extracted from the images^39^.

The severity of NAFLD is correlated with the hepatocellular fat content and biochemical dysfunction. In general, liver echogenicity increases with the severity of NAFLD. The high echogenicity of NAFLD is correlated not only with the total lipid content but even more strongly with the histomorphological distribution of fat deposits^40^. Ultrasonic tissue characterization involves the ultrasound-based assessment of quantitative information about the characteristics of biological tissues. Information about texture, attenuation, and backscattering can reflect the tissue structure and signal absorption and scattering in ultrasonic tissue characterization^41^. We hypothesize that ultrasonic tissue characterization can not only reflect the histopathological quantification of NAFLD, but also be used to detect small changes in the characteristics of liver fat. Therefore, this study performed an integrated analysis of texture, attenuation, and backscattering features to provide information about their usefulness in assessing disease severity in patients with NAFLD, and explored the relationship between the ultrasound findings and the biochemical characteristics of patients (Fig. 1).

## Results

In total, 394 subjects comprising 151 men and 243 women aged 40.5 ± 11.3 years (mean ± standard deviation) were recruited. The overall prevalence rates of mild and severe NAFLD were 32.2% and 26.9%, respectively (Table 1).

**Figure 2:**
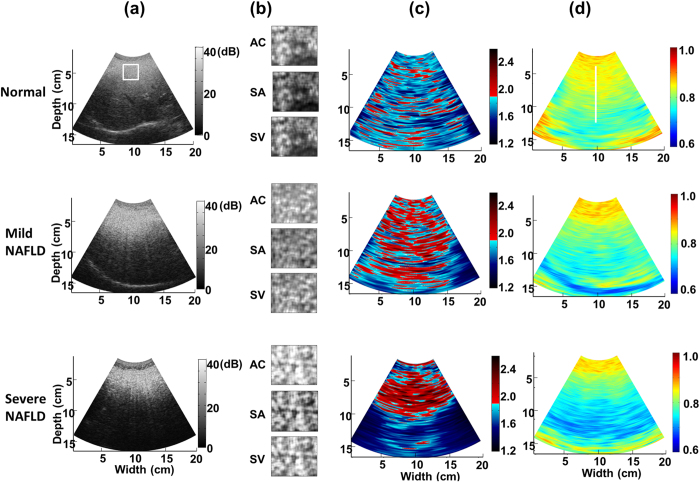
Different types of images of a normal liver and livers with mild and severe NAFLD: (**a**) ultrasound B-mode images, (**b**) texture-feature images, (**c**) ultrasound signal-to-noise ratio (SNR) images, and (**d**) ultrasound center-frequency downshift (CFDS) images. The white square and white line delineating the region of interest were manually traced by the physician. AC: autocorrelation; SA: sum average; SV: sum variance.

Figure 2(a) shows ultrasound B-mode images obtained from a normal liver and livers with mild and severe NAFLD. An increased severity of NAFLD was associated with increased echogenicity of the liver, increased ultrasound signal attenuation, and decreased definition of the diaphragm. The texture-feature images in Fig. 2(b) reveal that when the echogenicity of the liver increased, the autocorrelation (AC), sum average (SA), and sum variance (SV) increased. The physician manually chose the region of interest (ROI; delineated in Fig. 2(a) by a white square) in the texture-feature image to calculate the average texture-feature-image pixel values (i.e., average AC, SA, and SV values) for the liver. An ROI with a size of 1 × 1 cm^2^ was selected at a depth of 5 cm from the skin surface along the center line of the image to sample the liver parenchyma alone, in order to ensure that no blood vessels or other focal hypo-/hyperechogenic regions were included. Finally, linear discriminant analysis (LDA) was applied to combine the average AC, SA, and SV values to obtain the LDA-texture index^42^.

Figure 2(c) exemplifies the notable differences in the ultrasound signal-to-noise (SNR) images between normal and pathological livers, with the amount of red shading being greater for the pathological liver than for the normal liver, suggesting that the backscattered-signal statistics of the former were closer to a post-Rayleigh distribution. Moreover, the amount of red shading in the ultrasound SNR images of the livers increased with the severity of NAFLD. However, the deeper areas in images of severe NAFLD appeared blue due to the attenuation being larger. We copied the ROI obtained from the ultrasound B-mode image to the ultrasound SNR image, and calculated the average SNR image pixel value inside the liver ROI.

Figure 2(d) shows the ultrasound center-frequency downshift (CFDS) images in which areas with larger attenuation appeared cyan-blue while areas with smaller attenuation appeared yellow-red. Yellow-red shading was predominant in the ultrasound CFDS image of the normal liver, and the amount of cyan-blue shading increased with the severity of NAFLD. Furthermore, the color changed gradually with depth, with a larger change in color over a smaller distance when the attenuation was larger. The CFDS values for each point along the line that the physician had manually selected in the ultrasound CFDS image (delineated in Fig. 2(d) by a white line) were stored together with the depth information. The influence of diffraction effects was neglected to assume that the slope of CFDS was constant along the selected depth. Linear regression was applied to measure the CFDS slope, with the data fitted by a straight line using least-squares approximation. The CFDS slope of this line served as a quantitative attenuation-related index.

**Figure 3:**
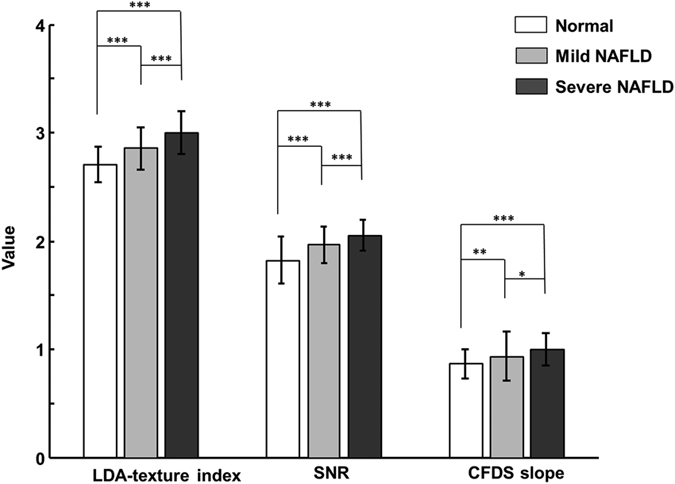
Mean and standard-deviation values of the linear discriminant analysis (LDA)-texture index, SNR, and CFDS slope for the normal, mild-NAFLD, and severe-NAFLD livers. *p < 0.05; **p < 0.01; ***p < 0.001. Data were assessed by an analysis of variance and trend test.

The mean and standard-deviation values of the LDA-texture index, SNR, and CFDS slope for the normal (*N* = 161), mild-NAFLD (*N* = 127), and severe-NAFLD (*N* = 106) livers are shown in Fig. 3. The LDA-texture indices for normal, mild-NAFLD, and severe-NAFLD livers were 2.710 ± 0.162, 2.858 ± 0.191, and 3.000 ± 0.197, respectively; the corresponding SNR indexes were 1.820 ± 0.216, 1.965 ± 0.169, and 2.053 ± 0.137, and the CFDS slope estimates were 0.866 ± 0.139, 0.935 ± 0.231, and 1.000 ± 0.146. The values of the LDA-texture index, SNR, and CFDS slope all increased significantly with the NAFLD severity (*p* for trend <0.05). These results indicate that the three parameters were useful for classifying the severity of NAFLD.

**Figure 4:**
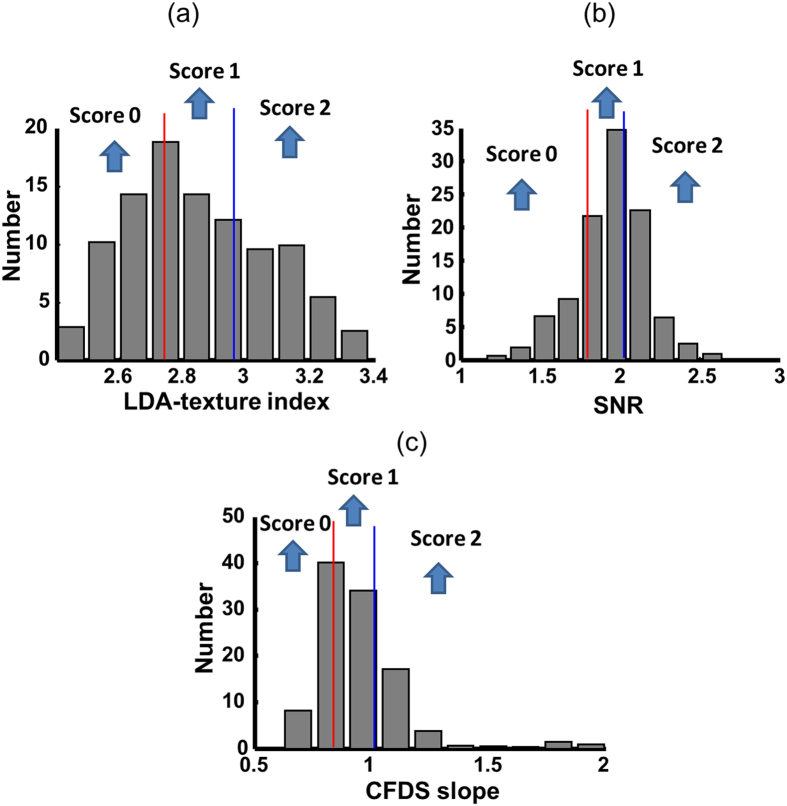
Distributions of (**a**) LDA-texture index, (**b**) SNR, and (**c**) CFDS slope in all of the subjects. The distributions were divided into tertiles. The first-/second-tertile cutoff points (red lines) of the LDA-texture index, SNR, and CFDS slope were 2.72, 1.85, and 0.84, respectively; and the corresponding second-/third-tertile cutoff points (blue lines) were 2.93, 2.00, and 0.95. The first-, second-, and third-tertile scores were coded as 0, 1, and 2, respectively.

Figure 4 represents the subjects divided into tertiles based on the values of the LDA-texture index, SNR, and CFDS slope to produce a quantitative diagnostic index (QDI). The first-, second-, and third-tertile scores were coded as 0, 1, and 2, respectively, and the scores for the three tests were summed to give a total QDI ranging from 0 to 6. The diagnostic performances were assessed in a separate investigation of normal versus NAFLD and normal versus severe NAFLD. We trained the QDI using the training data (normal liver = 126, mild NAFLD = 100, and severe NAFLD = 88) and applied it to the test data (normal liver = 35, mild NAFLD = 27, and severe NAFLD = 18) to assess the utility of the QDI. Table 2 lists the areas under the receiver operating characteristic curves (AUCs) for the QDI to identify NAFLD and severe NAFLD. For the training data, the AUCs of NAFLD and severe NAFLD were 0.81 (95% confidence interval [CI] = 0.77–0.86) and 0.84 (95% CI = 0.79–0.89), respectively, while the corresponding values for the test data were 0.73 (95% CI = 0.61–0.84) and 0.81 (95% CI = 0.67–0.93) (Table 2). In a multinominal logistic model, the QDI could distinguish severe NAFLD (odds ratio [OR] = 1.83, 95% CI = 1.52–2.2) and normal liver (OR = 0.62, 95% CI = 0.53–0.72) from mild NAFLD.

Table 3 indicates that the QDI was significantly correlated with the waist circumference, body mass index (BMI), fasting plasma glucose (FPG), total cholesterol (TCHO), triglycerides (TG), high-density lipoprotein cholesterol (HDL-C), low-density lipoprotein cholesterol (LDL-C), insulin, and a homeostasis model assessment of insulin resistance (HOMA-IR) in all subjects (*p* < 0.001). Moreover, in the subjects without NAFLD based on the US-FLI, the waist circumference, BMI, TCHO, TG, and LDL-C were still significantly associated with the QDI (*p* < 0.05).

## Discussion

In this study we found strong correlations between the parameters used to calculate the QDI and the severity of NAFLD. The AUCs of the QDI for diagnosing NAFLD and severe NAFLD were both higher than 0.81 for the training data. These values were slightly lower for the test data, because we randomly selected another set of subjects for the test data to avoid obtaining an overoptimistic classification performance. This meant that the performance for the test data was affected by the sample size. Although texture, backscattering and attenuation coefficients have been used to diagnose NAFLD, there is a trade-off between sensitivity and specificity when using a single parameter or combining the same types of physical parameter to detect NAFLD. The usefulness of a single parameter depends on the physical characteristics it represented, whereas combining the same physical parameters produces information overlap within the same physical dimension. The optimal types of feature parameters combined with each other are dependent on the physical meaning of each used parameter to some degree. This study demonstrated that combining texture, backscattering, and attenuation features can reflect the severity of NAFLD.

The formation of intracellular fat globules in NAFLD dramatically increases the echogenicity. We therefore used a parametric imaging method based on texture features of the gray-level co-occurrence matrix (GLCM) to characterize liver tissues^43^. Texture-feature imaging can preserve the local and global texture information, thus decreasing texture-analysis errors due to artifacts. The AC mirrored the roughness of the liver tissue, a higher SA indicated a B-mode image that was more homogeneously bright, and a higher SV meant that the echogenicity differed more from the global mean. Due to the AC, SA, and SV having the same physical dimensions, the linear combination of these parameters was used in LDA. It was found that high estimates of LDA-texture indices were associated with the more-severe form of NAFLD.

The Rayleigh distribution has been used to characterize the statistical distribution of backscattered-signal envelopes in normal liver tissue^37^,^44^,^45^. The statistical parameters of the homodyned K distribution or the Nakagami distribution have also been used to explore the relationship between the backscattered-signal statistics and the liver with NAFLD in rabbits and rats^46^,^47^. The scatterers in a liver with NAFLD will not necessarily be developed fully, which will result in a non-Rayleigh statistical model. The backscattered-signal envelopes will conform to Rayleigh statistics with an SNR equal to 1.91, so the simplest way to quantify this model is to use the SNR^37^,^48^. This study used ultrasound SNR imaging to analyze the distribution of ultrasound backscattered-signal envelopes. The concentration of fatty droplets is higher in NAFLD tissue than in normal liver tissue, which means that the arrangement and concentration of scatterers are changed so as to produce backscattered-signal envelopes that are more consistent with a post-Rayleigh distribution (i.e., higher SNR estimates). Therefore, the mean SNR estimates were found to increase significantly with the severity of NAFLD.

The measured attenuation corresponds to the summation of energy losses due to reflection, scattering, and absorption. Pauly and Schwan showed that scattering made only a very small contribution to the attenuation in normal livers, but that the scattering from fat droplets contributed significantly to the attenuation in fatty livers^49^. In addition, Kanayama *et al*. reported that the number of lipid droplets and their size in liver tissue can greatly contribute to energy absorption during ultrasound propagation^38^. Hence, we used the ultrasound CFDS imaging method to assess the local attenuation characteristics. The steeper CFDS slope in NAFLD was due to increased attenuation, because the fat itself absorbed ultrasound energy and fat droplets in the liver induced scattering. Therefore, a positive correlation between fatty infiltration of the liver and the CFDS slope was demonstrated. The estimated values of attenuation coefficients in previous studies were different because of different ultrasound imaging platforms and system settings, and different dataset. For example, Taylor *et al*. reported that the average attenuation coefficients in a normal liver and a fatty liver were 0.52 dB/cm/MHz and 0.77 dB/cm/MHz, respectively^50^. Lin *et al*. showed an increase from 0.71 to 1.22 dB/cm/MHz depending on the severity of fatty infiltration^51^. Lu *et al*. showed the mean attenuation at 3 MHz was 2.54 dB/cm in fatty livers compared to 1.66 dB/cm in healthy patients^30^. Fujii *et al*. represented the attenuation coefficients for the normal livers to be 0.59 dB/cm/MHz on average and that for the fatty livers to be 0.80 dB/cm/MHz on average^52^. Nevertheless, unanimous agreement was the attenuation coefficient increasing with the amount of liver fat increasing.

The relationship between NAFLD and metabolic syndrome is becoming increasingly recognized. Approximately 90% of patients with NAFLD have more than one feature characteristic of metabolic syndrome, placing NAFLD as the hepatic representation of metabolic syndrome^53^. Additionally, the presence of metabolic syndrome predicts a higher risk for the development of NAFLD^54^. The present study found that the QDI was significantly correlated with the extent of NAFLD as assessed in anthropometric and biochemical tests related to either insulin resistance or hepatocellular function. In particular, for the subjects with a US-FLI of 0 or 1, who have no NAFLD symptoms in a normal US-FLI examination, there appeared to be a positive correlation between the QDI and the features of metabolic syndrome. Namely, although visual criteria of hepatic echogenicity for diagnosing NAFLD were absent in these subjects, small changes in their liver fat were detected by the QDI and were associated with metabolic disorders. Arulanandan *et al*. demonstrated that larger amounts of liver fat are associated with an increased prevalence of the metabolic abnormalities independent of nonalcoholic steatohepatitis^55^. This supports that quantifying the amount of liver fat might have utility in predicting metabolic and cardiovascular risks. Furthermore, the QDI was significantly associated with metabolic factors. These results were similar to those of other studies that used MR imaging to quantify liver fat^56^,^57^. Above all, the QDI can potentially reflect the amount of liver fat when determining the severity of NAFLD and for predicting the risk for NAFLD development. Because the parameters used to calculate the QDI are related to various acoustic characteristic of a liver tissue, they reflect very useful information about the scatterer properties of a liver tissue.

There were several limitations to our study. First, the US-FLI rather than a histological examination was used as the reference. However, although calculating the US-FLI might be subjective, it was easily applicable to and correlated with histology parameters. Second, the influence of diffraction effect was neglected in estimating the CFDS slope, but the diffraction effect could affect the spectrum of backscattered signals. Third, the values of the LDA-texture index, SNR, and CFDS slope seemed likely to be affected by estimating errors by using the analytical method, the selected ROI, and the system settings. Fourth, the choice of tertile cutoff points for the QDI was based on the sample distribution. Fifth, ultrasound scanning is operator-dependent, and the evaluated parameters used to calculate the QDI would be dependent on the selected scanning slice.

While the severity of NAFLD was graded according to the US-FLI, we could not distinguish patients with pure NAFLD from those with combined NAFLD and inflammation/fibrosis. A previous study found that the US-FLI is strongly correlated with the steatosis percentage, but it was not correlated with fibrosis, since increased liver echogenicity in an ultrasound examination does not reflect the degree of fibrosis^28^. Several studies have found that the accumulation of more fat in the liver increases the likelihood of histological findings compatible with hepatic inflammation/fibrosis^33^,^50^,^58^. Taylor *et al*. found that a large attenuation was associated with fatty infiltration, whereas fibrosis has little effect on attenuation^50^. Gaitini *et al*. also reported that any combination of pathology with inflammation or fibrosis, even when severe NAFLD was present, altered the ultrasound backscattering properties and lowered the classification of the pathology in normal livers^33^.

Ultrasound elastography is well known to be useful in identifying NAFLD with fibrosis^59^,^60^,^61^. The velocity of the shear wave as measured by ultrasound elastography is related directly to tissue stiffness, with a harder tissue resulting in faster shear-wave propagation. For this reason, it would be interesting to integrate ultrasound elastography with the parameters used to calculate the QDI in order to separate the influences of fat and fibrosis in liver tissue. Another potential issue is to combine information from multiple images so as to reduce the uncertainty associated with analyzing only a single image. Most importantly, the liver biopsy and MR spectroscopy are still necessary to confirm the diagnosis and to explore the performance of QDI in future. Reproducibility and stability of the QDI in different ultrasound systems will be investigated.

In conclusion, the QDI is a novel quantitative scoring system that can be used to diagnose NAFLD. The QDI can reflect the acoustic characteristics of a liver to detect small changes in liver fat based on the following parameters used to calculate the QDI extracted from ultrasound B-mode images and backscattered signals: the LDA-texture index can supply information on the microstructure and macrostructure of the liver tissue, the SNR reflects the properties of scatterers within the liver, and the CFDS slope reflects the composition and biochemical environment of the liver. Furthermore, combining these three features should be functionally complementary and hence synergistic, and can improve the ability to grade the severity of NAFLD.

## Materials and Methods

### Ethics statement

The research protocol used in this study was approved by the Institutional Review Board of National Taiwan University Hospital (approval number, IRB#201210012RIC), and all subjects signed written informed consent. This study was performed in accordance with the Helsinki Declaration and Good Clinical Practice.

### Subject enrollment, anthropometric, and biochemical tests

All subjects were surveyed in detail using a standardized questionnaire, with exclusion criteria of excessive alcohol use (daily alcohol consumption of >20 g for women and >30 g for men), chronic liver disease (including chronic hepatitis carrier, autoimmune, drug-induced, vascular and inherited hemochromatosis and Wilson disease). The exclusion criteria were investigated at recruiting participants. There were 394 adults older than 20 years enrolled. Anthropometric and metabolic data were collected by routine physical examinations. BMI was calculated as the body weight divided by the height squared. The waist circumference was measured midway between the costal margin and iliac crests. FPG, TCHO, HDL-C, LDL-C, TG, and insulin were measured after 8 hours of overnight fasting. Insulin resistance was measured using HOMA-IR based on an iterative structural model simulating physical processes, which was developed by the Diabetes Trials Unit at the Oxford Center for Diabetes, Endocrinology, and Metabolism (available at http://www.dtu.ox.ac.uk/homacalculator).

### Ultrasound protocol

Ultrasound measurements were performed by three research physicians with more than 20 years of experience of performing liver ultrasound investigations. Before participating in the study, all of the physicians discussed the protocol of the ultrasound scan, including determining the US-FLI and the sequence used to obtain images of the liver. All of the operators agreed on a standard procedure to follow during the examination procedure. The subjects were imaged using a clinical ultrasound scanner (Model 3000, Terason, Burlington, MA, USA) equipped with a 3.5-MHz curved array transducer (Model 5C2A, Terason) to acquire raw RF data consisting of 128 scan lines of backscattered signals. The pulse length of transmission ultrasound was 1 mm and the −6 dB bandwidth of the transducer ranged from 2 to 5 MHz. Ultrasound RF signals were internally digitized at a sampling rate of 12 MHz. Each scan line was then demodulated using the Hilbert transform to obtain the envelope image, and the B-mode image was formed based on the logarithm-compressed envelope at a dynamic range of 40 dB. The axial and lateral resolutions were 0.6 mm and 1 mm, respectively. The focal zone was adjusted to the center of the liver to reduce the effect of beam diffraction. The time gain compensation sliders were aligned vertically in the central position, so the intensity of backscattered signals was not compensated along the depth. The instrument settings were standardized when imaging each subject, including identical gain and other relevant parameters.

The severity of NAFLD was evaluated according to a semiquantitative US-FLI ranging from 0 to 8 and composing five indicators: (1) presence or absence of liver-kidney contrast, graded as absent (score of 0), mild or moderate (score of 2), or severe (score of 3); (2) presence or absence of posterior attenuation of the ultrasound beam (score of 1 or 0), (3) presence or absence of vessel blurring (score of 1 or 0), (4) difficulty visualizing the gallbladder wall (score of 1) and difficulty visualizing the diaphragm (score of 1), and (5) area of focal sparing (score of 1)^27^,^28^. The subjects were divided into three groups of NAFLD severity according to the US-FLI: normal liver (US-FLI = 0 or 1), mild NAFLD (US-FLI = 2 or 3), and severe NAFLD (US-FLI ≥ 4)^27^,^28^.

### Texture analysis by texture-feature imaging

The GLCM was widely employed to represent texture information, including the orientation and distribution of the echo patterns in ultrasound B-mode images^33^,^62^,^63^. The texture analysis was based on a GLCM derived from the angular relationship between neighboring pixels as well as the distance between them^43^,^62^,^63^. Let represent the number of transitions between gray levels *i* and *j* to describe *P*(*i*, *j*|*d*, *θ*) the frequency of occurrence of two pixels that are separated by distance *d* in direction *θ*. Normalizing *P*(*i*, *j*|*d*, *θ*) provided the relative frequency or the probability of gray-level transitions^64^:


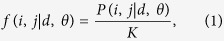


where *K* is the total number of gray-level transitions in the GLCM. The gray levels are quantized at 8 bits in the imaging system, and hence we used the full gray-level scale to estimate the texture features. In addition, possible directional bias was avoided by using four matrices obtained in different directions (*θ* = 0°, 45°, 90°, and 135°). To reduce the computation time, *d* was set 1.

Three texture-feature parameters described by Haralick *et al*. were selected that comprised typical characteristics of texture analysis^64^. The AC indicated the linear relationship between the gray levels of pixel pairs, the SA quantified the homogeneous brightness or darkness of the image, and the SV described the degree of heterogeneity and was closely related to the standard deviation. These parameters were calculated as follows^64^:


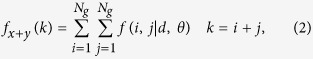



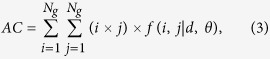



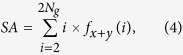



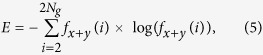



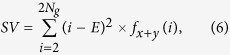


where *N*_*g*_ is the number of gray levels in the image.

The stable parenchymal B-mode image of the liver with no bowel or ribs shadowing was selected. To implement texture-feature imaging, a 3 × 3 mm^2^ sliding window was applied to the B-mode image to analyze each local texture feature, which involved calculating the average values estimated for the four matrices in different directions^43^. Note that the sliding window can be larger than the system resolution and satisfied the criterion to estimate the parameters. The window was moved through the entire gray-level B-mode image in steps of one pixel, with the local texture feature assigned as the new pixel located at the center of the window each time. This process yielded the texture-feature image as the map of texture-feature values.

### Backscattered-signal statistics in ultrasound SNR imaging

The SNR was analyzed as a backscattering parameter and depended on the density of scatterers. The statistics of backscattered signals tend to conform to the Rayleigh distribution when the numbers of scatterers in the resolution cell of the ultrasound transducer was higher than 10, corresponding to an SNR of 1.91 for the backscattered-signal envelopes. The SNR was defined as^37^,^65^


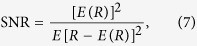


where *R* and E(·) are the backscattered-signal envelope and the statistical mean, respectively.

The square sliding window (3 × 3 mm^2^) was moved through the entire envelope image in steps of one pixel, with the local SNR assigned as the new pixel located at the center of the window each time. This process yielded the ultrasound SNR image as the map of SNR values. The SNR varied from 0 to 1.91, at which point the statistics of the backscattered-signal envelope changed from a pre-Rayleigh to a Rayleigh distribution, and the backscattered-signal statistics conformed to a post-Rayleigh distribution when the SNR was larger than 1.91. A pseudocolor scale was applied to present the information in the ultrasound SNR image. SNRs smaller than 1.91 were assigned blue shading that changed from dark to light with increasing value, signifying that the backscattered-signal envelope conformed to various degrees of a pre-Rayleigh distribution. An SNR of 1.91 was shaded white to indicate the Rayleigh distribution, and SNRs larger than 1.91 were assigned red shading that changed from dark to light with increasing value, indicating backscattered-signal statistics conforming to various degrees of a post-Rayleigh distribution.

### Attenuation measurement in ultrasound CFDS imaging

Since the tissue attenuation is both frequency and depth dependent, the frequency contents of the transmitted sound waves change as they propagate through tissue^66^,^67^,^68^. The ultrasound attenuation coefficient is determined by measuring the attenuations of different frequency components in soft tissue. The attenuation is larger for higher frequency components, and so the power spectrum of echo signals obtained using short-time Fourier analysis reveals the variation of CFDS with depth^67^,^68^. Assuming a Gaussian-shaped transmit pulse whose variance is invariant with depth, the correlation between the slope of CFDS versus depth and attenuation coefficient (*β*) is given by^68^


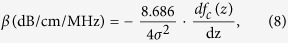


where *z* is the distance of the ROI from the transducer and *σ*^2^ is the variance of the transmit pulse. Variable *f*_*c*_(*z*) is the center frequency of the power spectrum at depth *z*^67^. Due to a linear dependence of the attenuation coefficient on frequency, the slope of CFDS versus depth was proportional to the attenuation coefficient.

The Fourier transform was used to calculate the block power spectrum by averaging spectra from the windowed segments within each block. The estimated center frequency was defined as the midpoint between the two sides of the full width at half maximum of the intensity in the block power spectrum. The CFDS was defined as


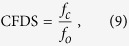


where *f*_*c*_ is the estimated center frequency and *f*_*o*_ is the center frequency of the transducer (3.5 MHz). The 3 × 3 mm^2^ sliding window was moved through the entire envelope image in steps of one pixel, with the local CFDS assigned as the new pixel located at the center of the window each time. This process yielded the ultrasound CFDS image as the map of CFDS values, which was presented using a pseudocolor scale. The lowest value was mapped to blue, the highest value was mapped to red, and the intervening values were mapped continuously onto values interpolated between these two colors in red-green-blue space.

### Statistical analysis

In this study, 314 subjects were allocated to a training data set for estimating quantitative parameters and another 80 subjects were allocated for testing the diagnostic performance of the QDI. The data were summarized as percentages for categorical variables and mean ± standard deviation values for continuous variables. Comparisons involving three or more groups were performed using an analysis of variance and trend test. The differences between each group were assessed by multiple comparisons using the Tukey-Kramer test. The performance of the QDI in discriminating NAFLD (US-FLI ≥2) and severe NAFLD (US-FLI ≥4) was evaluated using the AUC. We also applied a multinominal logistic regression model with mild NAFLD as a reference to assess whether the QDI can be used to differentiate the different NAFLD severities. The relationships between the QDI and anthropometric and biochemistry parameters were quantified using Pearson correlation coefficients. A difference was assumed to be statistically significant when the *p* value was less than 0.05.

## Additional Information

**How to cite this article**: Liao, Y.-Y. *et al*. Multifeature analysis of an ultrasound quantitative diagnostic index for classifying nonalcoholic fatty liver disease. *Sci. Rep*. **6**, 35083; doi: 10.1038/srep35083 (2016).

## Figures and Tables

**Figure 1:**
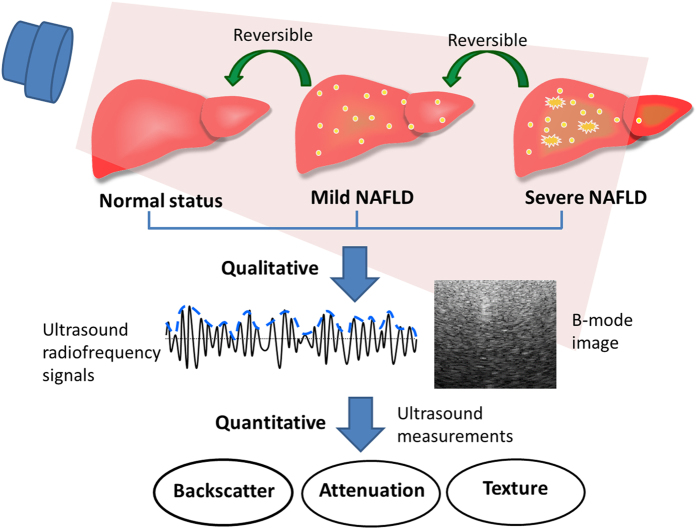
Illustration of ultrasound quantitative measurement method for the diagnosis and staging of patients with nonalcoholic fatty liver disease (NAFLD).

**Table 1:** Basic characteristic of the study subjects (*N* = 394).

Variable	*N* (%)	Mean ± SD	Range
Men	151 (38.3)		
Age (years)		40.5 ± 11.3	(20–72)
Anthropometric variables			
Waist circumference (cm)		81.1 ± 11.2	(55–120)
BMI (kg/m^2^)		24.1 ± 4.6	(14.9–43.7)
Biochemistry parameters			
FPG (mg/dL)		87.7 ± 17.6	(58–272)
TCHO (mg/dL)		192.9 ± 35.5	(101–320)
TG (mg/dL)		112.4 ± 90.3	(25–888)
HDL-C (mg/dL)		57.3 ± 15.8	(25–120)
LDL-C (mg/dL)		120.8 ± 32.5	(47–248)
Insulin (μU/mL)		9.10 ± 8.16	(2–84.4)
HOMA-IR		1.17 ± 1.00	(0.26–10.2)
NAFLD parameters			
US-FLI		2.23 ± 2.45	(0–8)
Mild NAFLD (US-FLI = 2 or 3)	127 (32.2)		
Severe NAFLD (US-FLI ≥ 4)	106 (26.9)		

Abbreviations: SD: standard deviation; BMI: body mass index; FPG: fasting plasma glucose; TCHO: total cholesterol; TG: triglycerides; HDL-C: high-density lipoprotein cholesterol; LDL-C: low-density lipoprotein cholesterol; HOMA-IR: homeostasis model assessment of insulin resistance; US-FLI: ultrasound fatty liver index.

**Table 2:** Area under the receiver operating characteristic curve (AUC) for NAFLD as defined by the US-FLI.

	Training data (*N* = 314)		Test data (*N* = 80)	
	OR (95% CI)	AUC (95% CI)	OR (95% CI)	AUC (95% CI)
NAFLD	2.12 (1.77–2.51)	0.81 (0.77–0.86)	1.60 (1.21–2.12)	0.73 (0.61–0.84)
Severe NAFLD	2.26 (1.86–2.73)	0.84 (0.79–0.89)	2.14 (1.39–3.29)	0.81 (0.67–0.93)

NAFLD was defined by US-FLI ≥2; severe NAFLD was defined by US-FLI ≥4Abbreviations: OR: odds ratio; CI: confidence interval.

**Table 3:** Correlation between the quantitative diagnostic index and metabolic factors in all subjects and in the subjects without NAFLD according to the US-FLI.

Variable	All subjects	Subjects without NAFLD by US-FLI
	*ρ*	*ρ*
Anthropometric variables		
Waist circumference (cm)	0.5134***	0.2935*
BMI (kg/m^2^)	0.4931***	0.2150*
Biochemistry parameters		
FPG (mg/dL)	0.2600***	0.1268
TCHO (mg/dL)	0.2035***	0.2239*
TG (mg/dL)	0.3960***	0.1885*
HDL-C (mg/dL)	−0.3591***	−0.0017
LDL-C (mg/dL)	0.2678***	0.2084*
Insulin (μU/mL)	0.2362***	0.0935
HOMA-IR	0.2519***	0.1009

*ρ*: Pearson correlation coefficient.

*p < 0.05; ***p < 0.001.
